# Parotid Enlargement as a Rare First Site of Manifestation in Extramedullary Acute Lymphoblastic Leukemia: A Case Report

**DOI:** 10.7759/cureus.65485

**Published:** 2024-07-27

**Authors:** Nursyamimi Mohmad Zaki, Azwan Halim Abdul Wahab

**Affiliations:** 1 Department of Otolaryngology, Head and Neck Surgery, International Islamic University Malaysia, Kuantan, MYS

**Keywords:** fine-needle aspiration cytology, parotid tumor, acute lymphoblastic leukemia, acute parotitis, extramedullary leukemia

## Abstract

Parotid neoplasm in children is very rare, and most of these tumors are benign. Parotid enlargement in children is usually caused by infection or inflammation. We report a case of a 12-year-old boy who presented with the initial manifestation of bilateral parotid enlargement. He complained of two weeks of parotid swelling, during which the tumor gradually increased in size as he battled a monthlong on-and-off fever. An intravenous antibiotic was administered, as the first diagnosis was infection. Imaging studies of the swelling displayed features of infection, which was not resolved by the antibiotic. Fine-needle aspiration cytology was initially planned to establish a diagnosis. However, due to other findings in the clinical examination, such as bilateral scrotal swelling with abnormal blood work, the child was referred to other specialists for further assessment. Eventually, he was diagnosed with B-cell acute lymphoblastic leukemia by a hematology team.

## Introduction

The most common causes of parotid enlargement in children are infections and inflammation [1]. Primary neoplasms of parotid glands are rare in pediatric age groups, but secondary malignancies have been reported in childhood leukemia survivors. Acute lymphoblastic leukemia (ALL) is the most common malignancy in children, comprising approximately one-third of all childhood cancers and having a survival rate of approximately 90% [2,3]. A few cases of the parotid glands being the site of involvement in relapses of acute myeloid leukemia (AML) and ALL have been reported, but bilateral parotid enlargement as the initial presenting manifestation is quite rare. A working diagnosis of childhood leukemia must be considered in managing a patient with parotidomegaly, especially if the patient exhibits abnormal blood work, to avoid any delay in the improvement of the prognosis.

## Case presentation

A 12-year-old Malay boy with no known medical illness and a complete record of immunizations first presented to the emergency department with complaints of bilateral parotid swelling that gradually increased in size over two weeks' duration. The swelling was associated with on-and-off pain and difficulty opening the mouth and chewing. It was also preceded by a history of an intermittent fever that began about one month prior to the presentation. The boy initially sought care from a general practitioner and was prescribed an oral antibiotic; however, the swelling persisted in spite of the antibiotic. Otherwise, the boy had no history of contact with others who were sick or had any constitutional symptoms. He was then referred to us for further assessment.

Upon examination, we found signs of mild pallor and trismus. The facial nerve examination was unremarkable. The parotid swelling was bilateral but larger on the right side than the left, measuring approximately 5 x 6 cm and 3 x 4 cm, respectively. The parotid swelling was tender upon palpation but firm in consistency; distended vessels but no punctum were seen on the swelling. There were two cervical lymph nodes on the right, measuring approximately 1 x 2 cm each. Intraoral examination revealed no discharge from the bilateral Stensen's duct. Further physical examination revealed bilateral scrotal swelling that measured approximately 3 x 2 cm in each testis, but the testes were not tender and exhibited no other changes.

The patient was then admitted and treated for bilateral parotitis with orchitis with intravenous administration of amoxicillin and clavulanic acid along with analgesics. He was also referred to the Urology Department for further assessment, and an urgent parotid ultrasound was scheduled. The test revealed features representing bilateral parotitis with early abscess formation.

**Figure 1:**
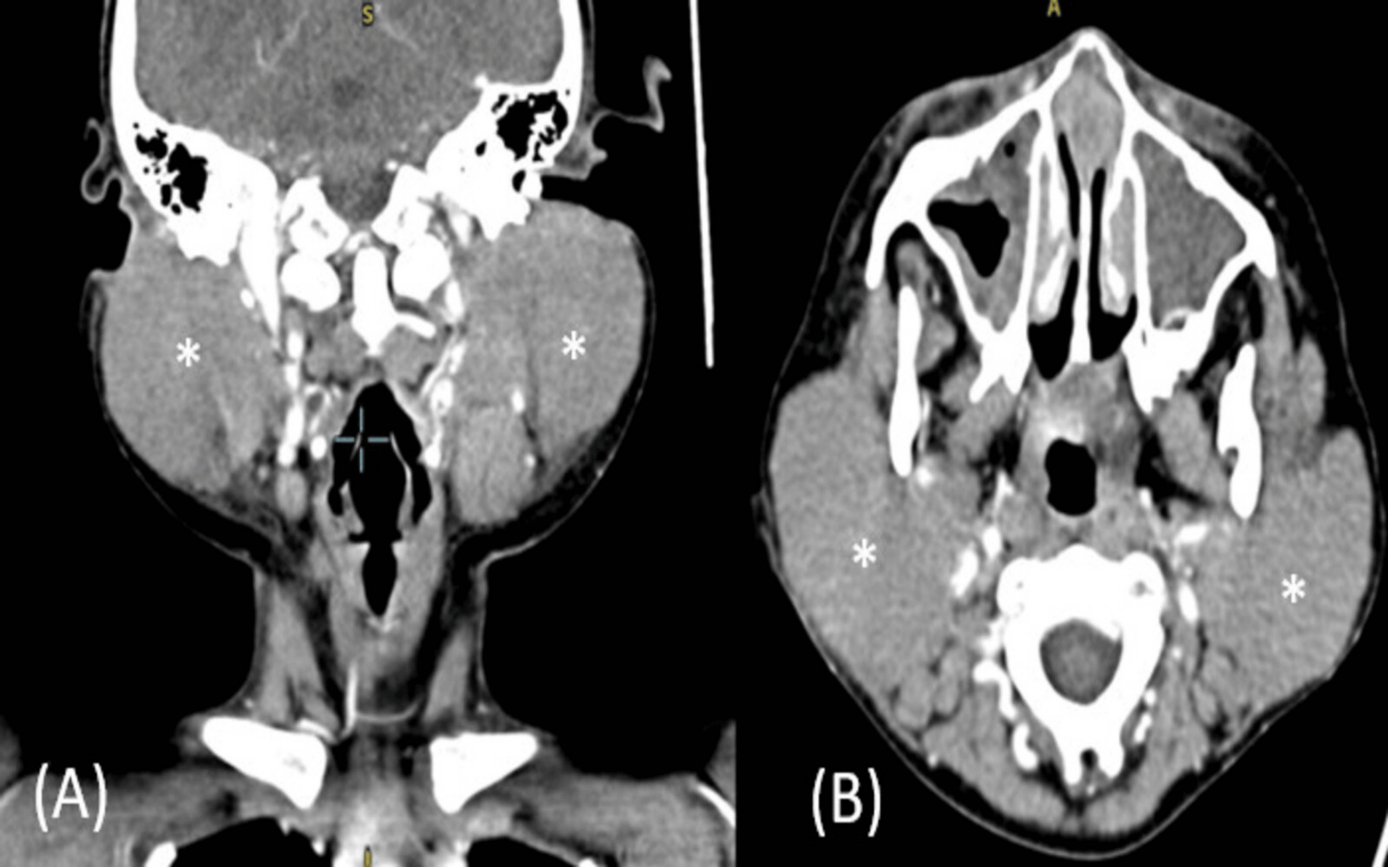
A CECT of the neck showed homogenously enhanced bilateral parotid glands and appeared bulky, but no focal hypodense area with peripheral enhancement was visible to suggest collection. (A) A CECT of the neck sagittal cut with asterisks showed bilateral parotid masses; (B) CECT of the neck axial cut with asterisks showed bilateral parotid masses. CECT: contrast-enhanced computed tomography.

No improvement was observed with the intravenous antibiotics, but an increasing trend of total white cells (9.9 x 10/L to 31.3 x 10/L) and a decreasing trend of hemoglobin (9.1 g/dL to 7.1 g/dL) were noted. The patient was then referred to a pediatric team to rule out hemophagocytic syndrome, while contrast-enhanced computed tomography (CECT) of the neck was done to look for new abscess formation and extension. However, the CECT revealed no collection in the parotid glands and overall features suggestive of bilateral parotitis (Figure 1).

Additionally, fine-needle aspiration cytology (FNAC) was conducted to look for other causes. The pediatric team suggested escalating the antibiotic to intravenous cefuroxime and ordering an urgent full blood picture, which then revealed leukocytosis with the presence of 66% blast cells. This was highly suggestive of acute leukemia, most probably ALL.

The FNAC was then abandoned, and the patient was referred to the hematology team. He underwent a bone marrow aspiration and trephine biopsy with immunophenotyping, which confirmed the presence of B-cell ALL. The hematology team ordered appropriate treatment, and the patient is currently in remission post-treatment.

## Discussion

Parotid enlargement in children is usually caused by infections, with mumps being the most common. Among other causes are *Staphylococcus aureus*, human immunodeficiency virus (HIV), *Mycobacterium tuberculosis*, echovirus, parainfluenza (types 1 and 3), Coxsackievirus A, adenovirus, and cytomegalovirus [4]. Parotid neoplasm in children is quite rare, and approximately 80% of the masses are benign. The incidence of malignant parotid in children is even rarer. The most common parotid malignancy is the mucoepidermoid cyst, followed by acinic cell carcinoma [5].

Childhood ALL is a cancer of the blood and bone marrow. It is the most common childhood malignancy and has a five-year survival rate exceeding 90% [6], but it can worsen quickly if it is not diagnosed and treated early. The most common clinical symptoms of ALL are usually a result of medullary involvement, including leukopenia, thrombocytopenia, and anemia, which prompt the patient to present with bleeding tendencies, fatigue, anorexia, malaise, bone pain, pallor, and fever. Extramedullary sites of manifestation include ocular, bone, renal, hepatic, abdomen, bladder, skin, oral, pericardium, and pancreas [7]. Parotid involvement has been reported in relapse cases of AML and ALL, but parotid gland involvement as the first manifestation of ALL is unusual. Moreover, the parotid gland has been reported to be a sanctuary site for relapses of AML, either in isolation or in association with the central nervous system [7,8]. It also remains unclear whether parotid gland involvement in ALL warrants aggressive local or systemic therapy, signifies a risk of a second malignancy, or may later act as a sanctuary site [8,9].

The differential diagnosis between a benign and a malignant parotid lesion cannot be established by simple physical examination but requires complementary diagnostic methods. FNAC is a safe and cost-effective method to aid in the diagnosis of salivary gland neoplasms. As a minimally invasive procedure that does not require anesthesia [10], FNAC can be done in an outpatient setting with a low risk of complications. Although it has become one of the most common initial tests to evaluate parotid masses, there remains some controversy regarding its effectiveness due to its low sensitivity in differentiating benign from malignant tumors [9].

Nevertheless, in pediatric lymphoid tumors, embryonal tumors, and vasoformative lesions, the accuracy of FNAC is decreased [8,9]. In our case, FNAC was planned initially to establish a diagnosis in view of uncorrelated imaging studies (both ultrasonography and CECT studies of the parotid revealed features of infection) and abnormal complete blood count results (leukocytosis with anemia). Subsequently, when the complete blood picture result indicated leukocytosis with the presence of 66% blast cells, which was highly suggestive of acute leukemia and most probably ALL, FNAC was abandoned, and the young patient underwent bone marrow aspiration and trephine with immunophenotyping, which confirmed the presence of B-cell ALL.

## Conclusions

The parotid gland is a rare extramedullary site for the initial presentation of ALL. However, a working diagnosis of ALL must be kept in mind by a physician or surgeon when treating a child who presents with parotid swelling but with an abnormal course of disease or blood investigation. ALL is the most common cause of malignancy in children, but it has a high survival rate if it is recognized and treated early. The diagnosis of parotid malignancies can be a challenge in treating a child with parotid swelling, as these malignancies are less common than infections and inflammation in the pediatric age group. It is crucial that physicians order an appropriate blood workup when treating a child with parotid swelling and draw on their intuition and vast medical knowledge to diagnose ALL, as doing so will improve the prognosis and survival rate of the patient.
